# Proinflammatory Effect of Carbon-Based Nanomaterials: In Vitro Study on Stimulation of Inflammasome NLRP3 via Destabilisation of Lysosomes

**DOI:** 10.3390/nano10030418

**Published:** 2020-02-27

**Authors:** Tereza Svadlakova, Frantisek Hubatka, Pavlina Turanek Knotigova, Pavel Kulich, Josef Masek, Jan Kotoucek, Jan Macak, Martin Motola, Martin Kalbac, Martina Kolackova, Radka Vankova, Petra Vicherkova, Andrea Malkova, Pavlina Simeckova, Yuri Volkov, Adriele Prina-Mello, Irena Kratochvilova, Zdenek Fiala, Milan Raska, Jan Krejsek, Jaroslav Turanek

**Affiliations:** 1 Institute of Clinical Immunology and Allergology, University Hospital Hradec Kralove and Faculty of Medicine in Hradec Kralove, Charles University, 50005 Hradec Kralove, Czech Republic; svadlakovat@lfhk.cuni.cz (T.S.); kolackovam@lfhk.cuni.cz (M.K.); vankovr@lfhk.cuni.cz (R.V.); petraavicherkova@gmail.com (P.V.); 2 Institute of Hygiene and Preventive Medicine, Faculty of Medicine in Hradec Kralove, Charles University, 50003 Hradec Kralove, Czech Republic; Malka8AR@lfhk.cuni.cz (A.M.); fiala@lfhk.cuni.cz (Z.F.); 3 Veterinary Research Institute, 62100 Brno, Czech Republic; hubatka@vri.cz (F.H.); knotigova@vri.cz (P.T.K.); kulich@vri.cz (P.K.); masek@vri.cz (J.M.); kotoucek@vri.cz (J.K.); simeckova@vri.cz (P.S.); milan.raska@upol.cz (M.R.); 4 Center of Materials and Nanotechnologies, Faculty of Chemical Technology, University of Pardubice, 53002 Pardubice, Czech Republic; Jan.Macak@upce.cz (J.M.); martin.motola@upce.cz (M.M.); 5 J. Heyrovsky Institute of Physical Chemistry of the Czech Academy of Sciences, 18223 Prague, Czech Republic; martin.kalbac@jh-inst.cas.cz; 6 Department of Clinical Medicine/Trinity Translational Medicine Institute (TTMI), Trinity College Dublin, D08 W9RT, Dublin, Ireland; yvolkov@tcd.ie (Y.V.); prinamea@tcd.ie (A.P.-M.); 7 Department of Histology, Cytology and Embryology, First Moscow State Sechenov Medical University, 119992 Moscow, Russia; 8 Institute of Physics, Czech Academy of Sciences, 18200 Prague, Czech Republic; krat@fzu.cz; 9 Department of Immunology and Institute of Molecular and Translational Medicine, Faculty of Medicine and Dentistry, Palacky University Olomouc, 77515 Olomouc, Czech Republic

**Keywords:** graphene platelets, carbon nanotubes, inflammasome NLRP3, cathepsin B, macrophages, THP-1

## Abstract

Carbon-based nanomaterials (C-BNM) have recently attracted an increased attention as the materials with potential applications in industry and medicine. Bioresistance and proinflammatory potential of C-BNM is the main obstacle for their medicinal application which was documented in vivo and in vitro. However, there are still limited data especially on graphene derivatives such as graphene platelets (GP). In this work, we compared multi-walled carbon nanotubes (MWCNT) and two different types of pristine GP in their potential to activate inflammasome NLRP3 (The nod-like receptor family pyrin domain containing 3) in vitro. Our study is focused on exposure of THP-1/THP1-null cells and peripheral blood monocytes to C-BNM as representative models of canonical and alternative pathways, respectively. Although all nanomaterials were extensively accumulated in the cytoplasm, increasing doses of all C-BNM did not lead to cell death. We observed direct activation of NLRP3 via destabilization of lysosomes and release of cathepsin B into cytoplasm only in the case of MWCNTs. Direct activation of NLRP3 by both GP was statistically insignificant but could be induced by synergic action with muramyl dipeptide (MDP), as a representative molecule of the family of pathogen-associated molecular patterns (PAMPs). This study demonstrates a possible proinflammatory potential of GP and MWCNT acting through NLRP3 activation.

## 1. Introduction

Over the past decades, nanomaterials have attracted great attention with C-BNM being among the most studied ones. Their extraordinary physicochemical properties, namely, tuneable electronic (e.g., band gap) and electrical properties (e.g., conductivity), thermal and chemical stability, and large surface area, make C-BNM appropriate candidates for a wide range of high-tech applications, as well as for biomedical applications [1,2,3,4,5,6]. Chemical industry is capable of producing C-BNM in large quantities and, therefore, their impact on the environment is inevitable. C-BNM, predominantly graphene oxide (GO) and carbon nanotubes (CNT), were tested for their potential use in nanomedicine as drug carriers. However, incomplete toxicology data together with an increasing demand on the production of these materials dictate the necessity for toxicological studies to answer questions about their safety with respect to guidance issued by FDA and EMEA.

An important concern relates to the C-BNM interaction with the immune system and their ability to induce an inflammation. Even though both CNT and graphene have been extensively investigated over the recent years, the results are still inconsistent and often contradictory. Asbestos-like shape of CNT leads to the assumption that both single and multi-walled carbon nanotubes (SWCNT and MWCNT) have a capacity to induce acute and chronic inflammation [7,8,9]. Depending on the shape, length, functionalization and presence of impurities, the mechanical disruption associated with oxidative stress, mitochondrial damage, and production of pro-inflammatory cytokines are considered principal mechanisms of cytotoxicity [10,11,12]. A similar situation could arise for graphene, the 2D layered nanomaterial, which can also be expected to cause mechanical damage to cell membranes via flat and sharp edges [13,14]. Many studies are focused on graphene oxide (GO), which is described as a promising nanomaterial in nanomedicine despite the fact, that many reports point to its potential cytotoxicity [15,16,17,18,19,20,21,22,23,24,25]. However, there are limited data on biological effects of other derivatives such as GP and graphene sheets to which humans are exposed during development, processing or manufacturing [26,27]. These non-biodegradable materials could pose a risk, especially for the respiratory system after exposure by inhalation [28]. It has been confirmed that GP, which are up to 25 μm in diameter, can be delivered beyond the ciliated airways and deposited in alveoli, where they can either persist in intercellular spaces, or are internalized by alveolar macrophages [29,30,31]. This may lead to inflammation, disruption of homeostasis, and subsequent fibrosis and tissue damage [28,32,33,34]. For example, multi-layered graphene platelets induced a substantial inflammatory response and cytotoxicity in rat lungs [33]. Another pulmonary in vivo study which compared six different surface modifications of GP showed increased oxidative stress and acute inflammation [35]. Moreover, positively-charged GP also showed significant inflammation characterized by the accumulation of neutrophil granulocytes [31]. On the other hand, single-layered graphene oxide platelets did not cause acute cytotoxicity or inflammation in a 3D human lung model and similarly, no acute toxicity was demonstrated after 28 days of in vivo exposure to multi-layered graphene platelets [29,36]. Obviously, a focus on deeper details on the mechanism of cytotoxicity and the inflammation of C-BNM is still necessary.

One of the key mediators of nanomaterials-induced inflammation could be activation of NLRP3 inflammasome [37]. Activation of NLRP3 is a complex process, which is evoked in response to infectious stimuli like whole pathogens or individual PAMPs, which escaped to the cytoplasm, as well as by cellular stress signals represented by sterile damage signals (DAMPs). Some studies focused on the assembly of this complex as the leading mechanism of pulmonary inflammation after exposure to all nanotubes and nanowire materials [38,39,40]. As a principal mediator of inflammasome assembly, activation of NADPH oxidase, which led to the oxidative burst and, subsequently, to lysosomal damage and a release of cathepsin B, was suggested [38]. In our previous study we found that nanodiamonds (ND) can disrupt lysosomal membrane and subsequently activate NLRP3 via cathepsin B activation pathway [6]. This leads to a hypothesis whether pristine graphene and its derivatives are also capable of induction of inflammation via the inflammasome pathway. Therefore, we used the same model of THP1-Null cells (derived from THP-1 human monocytic cells). This cell line represents a well-established in vitro system for the studies of canonical NLRP3 inflammasome pathway, as it expresses high levels of NLRP3, ASC and pro-caspase 1. Upon activation of caspase-1 in THP1 cells, bioactive IL-1β is detected with high sensitivity and specifity by HEK-Blue™IL-1 β reporter cell-based assay. In this study, we assessed MWCNT and two types of GP by their potential to penetrate the cells, affect cell viability, destabilise lysosomes, and activate inflammasome NLRP3 by canonical and non-canonical pathways. All these nanomaterials are assumed to cause chronic inflammation. For this reason, we focussed on their ability to induce NLRP3 stimulation as a main mechanism of their proinflammatory effects. We demonstrate here that GP and MWCNT possess proinflammatory potential executed both via canonical and alternative pathways.

## 2. Materials and Methods 

### 2.1. Carbon-Based Nanomaterials Characterization

Two different types of GP and MWCNT were used in this study. GP1 was purchased from PlasmaChem GmbH (Berlin, Germany) as a powder (product number PL-P-G750). According to the manufacturer’s specifications, the particle size was up to 2 μm and the thickness of graphene sheets was 1–4 nm. GP2 was kindly donated by CRANN (the Centre for Research on Adaptive Nanostructures and Nanodevices), Trinity College Dublin (Dublin, Ireland) as a powder. MWCNT were purchased from Sigma-Aldrich (St. Luis, MO, USA) as a powder (product number 659258).

All three types of materials were thoroughly investigated for composition, structure, and thermal stability. Results of the X-ray diffractometry, energy dispersive X-ray spectroscopy, Raman spectroscopy and thermogravimetric analyses are introduced and discussed in detail in the Supplementary Information. Briefly, all obtained physico-chemical characterisation results were in line with specifications of the producers of these materials.

### 2.2. Preparation of Suspensions

Stock suspensions of GP at a concentration 250 µg/mL were prepared by dispersing powders in 0.02% sodium cholate, followed by sonication using a sonic probe (QSonica, Q700 ultrasonic processor with a 1/4" microtip probe) for 30 min with 65% of amplitude. The average shape and size were assessed by transmission electron microscopy (TEM, Philips 208 S Morgagni, FEI) at an accelerating voltage of 80 kV and by scanning electron microscopy (SEM, Magellan 400L, FEI). The hydrodynamic diameters (D_H_) of GP were determined using Zetasizer Nano-Ultra (Malvern Panalytical Ltd, Malvern, UK). Measurements were provided in several dilution of stock solutions in Milli-Q water and cell culture media containing of 10% FBS. 

Stock suspension of MWCNT at a concentration 500 µg/mL was prepared by dispersing powder in 0.02% sodium cholate and sonicating using QSonica (Melville, NY, USA), Q700 5 min with 55% of amplitude. The average length and diameters of all used materials were assessed by TEM at an accelerating voltage of 80 kV and by SEM.

### 2.3. Zeta Potential

Zeta potential measurements were performed on a Zetasizer Nano ZSP instrument equipped with MPT-2 Titrator (Malvern Panalytical Ltd, Malvern, UK). The instrument is using a He–Ne laser (wavelength of 633 nm) and detector angles of 173° and 13°. Data were recorded and analysed using Zetasizer Software v7.11. Prior to all zeta potential measurements, all C-BNM were diluted in 10 mM Na_2_HPO_4_ solution with pH 7.2. ζ-potential values were calculated using the Smoluchowski equation. Each data value represents an average of three measurements.

### 2.4. Cell Culture 

Human cell line THP-1 was purchased from The European Collection of Authenticated Cell Cultures (ECACC, Salisbury, UK). All human reporter cell lines, THP1-null, THP1-defNLRP3, THP1-defASC and HEK-Blue™ IL-1β were purchased from InvivoGen (San Diego, CA, USA). THP-1 were maintained in RPMI 1640 media without phenol red (Corning, NY, USA) supplemented with 10% heat inactivated ultra-low endotoxin fetal bovine serum (FBS_LE_; Biosera, France), 2 mM L-alanyl-L-glutamine (GlutaMAX; Life Technologies, Carlsbad, CA, USA), 1 mM sodium pyruvate (Life Technologies, Carlsbad, CA, USA), 10 mM HEPES (Sigma-Aldrich, St. Luis, MO, USA), 0.05 mM 2-mercaptoethanol and with penicillin (100 U/mL) and streptomycin (100 µg/mL) (Sigma-Aldrich, St. Luis, MO, USA). THP1-null, THP1-defNLRP3 and THP-1 defASC were maintained in RPMI 1640 media supplemented with heat inactivated 10% foetal FBS_LE_, 25 mM HEPES, Normocin (100 µg/mL; InvivoGen, San Diego, CA, USA) and selection antibiotic Hygromycin B Gold (200 µg/mL; InvivoGen, San Diego, CA, USA). HEK-Blue™ IL-1β were maintained in Dulbecco’s modified Eagle’s High Glucose medium (DMEM; Sigma-Aldrich, St. Luis, MO, USA) supplemented with 10% FBS_LE_ and with selection antibiotics Hygromycin B Gold (200 µg/mL) and Zeocin (100 µg/mL; InvivoGen, San Diego, CA, USA). All cells were incubated in a humidified atmosphere of 5% CO_2_ at 37 °C.

Peripheral blood samples were obtained from healthy volunteers after an informed consent and approval by the Ethics Committee, University Hospital Hradec Kralove, Sokolska 581, 500 05 Hradec Kralove (reference number 201902 S22P), Czech Republic. Peripheral monocytes were isolated from whole blood using RosetteSep™ Human Monocyte Enrichment Cocktail (STEMCELL Technologies Inc.,Vancouver, Canada) according to manufacturer’s protocol. The purified monocytes were verified by flow cytometry (~94%) and maintained in RPMI 1640 supplemented with 20% human autologous serum and Primocin™ (100 µg/mL, InvivoGen, San Diego, CA, USA). The cells were incubated in a humidified atmosphere of 5% CO_2_ at 37 °C.

### 2.5. Cell Viability and Plasma Membrane Integrity

Cell viability was assessed through lactate dehydrogenase (LDH) assay. THP-1 cells were seeded in flat bottom 96-well plates at the density 4 × 10^4^ cells per well and treated with phorbol 12-myristate 12-acetate (PMA; 25 ng/mL, Sigma-Aldrich, St. Luis, MO, USA) for 72 h. After differentiation, cells were washed and exposed to increasing concentration of GP and MWCNT in media (5–60 µg/mL) for 24–72 h. Cells with no exposure and cells exposed to sodium cholate were used as controls. Supernatants were centrifuged at 10,000× *g* for 10 min to eliminate GP and MWCNT and transferred into a new flat bottom 96-well plate. The LDH assay was performed according to the manufacturer’s protocol. Absorbance was measured in a microplate spectrophotometer Synergy HTX (Biotek, Bad Friedrichshall, Germany) at 490 nm, with 690 nm set as the reference wavelength.

### 2.6. Mitochondrial Potential Detection

PMA differentiated THP-1 cells exposed to all C-BNM samples were washed with a phosphate buffered solution (PBS) and subsequently treated with cell permeable probe tetramethylrhodamine ethyl ester (TMRE, 750 nM, Sigma-Aldrich, St. Luis, MO, USA) for 30 min. TMRE intensity fluorescence was determined by the microplate spectrophotometer with excitation/emission wavelengths of 549/575 nm. GP and MWCNT were incubated with fluorescence probes to determine possible interferences. TMRE-stained mitochondria were also observed using a holotomographical microscope Nanolive 3D Cell Explorer – fluo with software STEVE version 1.6.3496 (Nanolive, Ecublens, Switzerland).

### 2.7. Intracellular Localization of C-BNM 

THP-1 cells were prepared as described above for the viability assays. Cells exposed to 25 and 50 µg/mL C-BNM were collected and fixed in 3% glutaraldehyde. GP and MWCNT samples were centrifuged and the pellet was rinsed in Milonig buffer, post-fixed in 1% OsO_4_ solution in Milonig buffer, dehydrated in 50%, 70%, 90%, 100% ethanol, embedded in Epon-Durcupan mixture (Epon 812 Serva, Heidelberg, Germany; Durcupan, ACM Fluka, Buchs, Switzerland) and polymerized at 60 °C for 72 h. Ultrathin (60 nm) sections were cut with glass knives on UC 7 ultramicrotome (UC 7, Leica, Vienna, Austria) and contrasted by 2% uranyl acetate and 2% lead citrate. The sections were examined using TEM (Philips 208 S Morgagni, FEI, San Jose, CA, USA).

### 2.8. Activation of NLRP3

THP1-null cells, as they express high levels of NLRP3, adaptor protein ASC (apoptosis-associated Speck-like protein with a caspase recruitment domain) and pro-caspase 1 were seeded in the flat bottom 96-well plates at density 360 × 10^3^ cells per well and primed with ultrapure lipopolysaccharide (LPS,1 µg/mL, Invivogen) for 3 h. Cells were subsequently washed and stimulated with C-BNM (5–60 µg/mL) and with sodium cholate as a control for 24–48 h. Collected supernatants were centrifuged at 10,000× *g* for 10 min to eliminate free C-BNM and transferred (50 µL) to new flat bottom 96-well plates. Mature (cleaved) IL-1β in supernatants was detected by cell-based assay using HEK-Blue™ IL-1β cells. HEK-Blue™ cells respond specifically to IL-1β. Binding of IL-1β to its receptor IL-1R on the surface of HEK-Blue™ allows sensitive specific detection of bioactive interleukins via colorimetric assay of enzyme activity of expressed reporter gene SEAP. SEAP was quantified using QUANTI-Blue™ a SEAP detection medium, which turns blue in its presence. THP1-defNLRP3 and THP-1 defASC cell lines, which are deficient of NLRP3 and ASC, respectively, were primed and exposed in the same way as the THP1-null cells and were used as negative controls. Absorbance was measured in a microplate spectrophotometer at 630 nm wavelength.

Supernatants from isolated monocytes exposed to all C-BNM (5–60 µg/mL for 24–48 h) were collected and centrifuged at 10,000× g for 10 min to get rid of non-internalised free GP and MWCNT and transferred (50 µL) to a new flat-bottom 96-well plate. LPS (100 ng/mL) was used as a positive control and the specific inhibitor MCC950 (Invivogen, Paris, France) was used as a verification of a specific NLRP3 inflammasome activation. Mature IL-1β in supernatants was detected by cell-based assay using HEK-Blue™ IL-1β cells. Absorbance was measured in a microplate spectrophotometer at 630 nm wavelength. 

### 2.9. Release of Cathepsin B

Detection of cathepsin B in THP1-null cells exposed to GP and MWCNT (30 µg/mL) was performed by cathepsin B detection kit (Enzo, LifeSciences, Farmingdale, NY, USA) according to the manufacturer’s protocol. CV-(RR)2 was used as a substrate for cathepsin B cleavage. As a positive control, cells were pre-treated by lysosomal disruptor Leu-Leu methyl ester hydrobromide LLME (100 µM, Sigma-Aldrich, St. Luis, MO, USA) for 2 h. Cells not exposed to GP and MWCNT were used as negative controls.

### 2.10. Inflammatory Cytokines Production

For cytokine detection in supernatants from exposed THP-1 cells and primary monocytes, ELISA kits for IL-6, TNF-α and IL-10 (R&D Systems, Minneapolis, MN, USA) human cytokines were used. Kits were used according to the manufacturer’s protocol.

### 2.11. Statistical Analysis

Data are expressed as mean values (*n*_tests_ = 3) ± standard deviation and are normalized to the control untreated cells (control). Differences have been considered significant for *p* values ˂ 0.05. Two-way ANOVA with Bonferroni post hoc test was performed, using GraphPad Prism™ software version 7.00 (GraphPad Software Inc., San Diego, CA, USA).

## 3. Results

### 3.1. C-BNM Characterization

**Figure 1:**
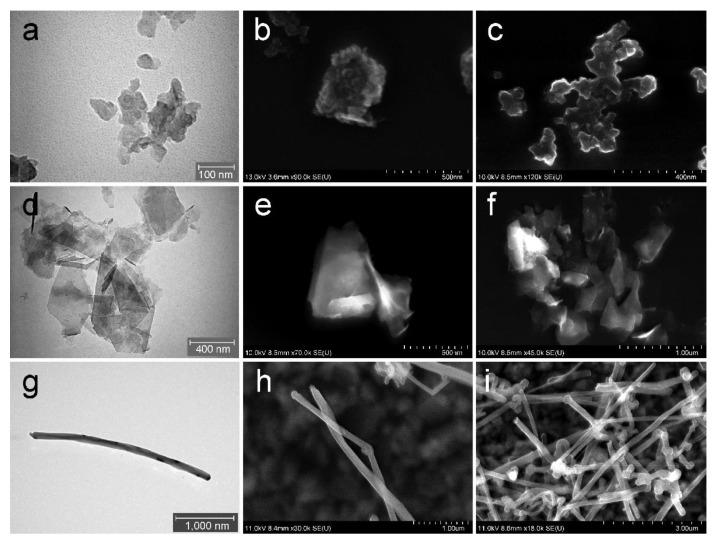
Characterisation of C-BNM by electron microscopy: (**a**) TEM and (**b**,**c**) SEM detail of the GP1 forming small aggregates; (**d**) TEM detail of the GP2; (**e**) SEM detail of a structure of GP2 single platelet and (**f**) forming clusters; (**g**) TEM detail of single MWCNT; (**h**) SEM detail of a structure of MWCNT with (**i**) forming clusters.

**Figure 2:**
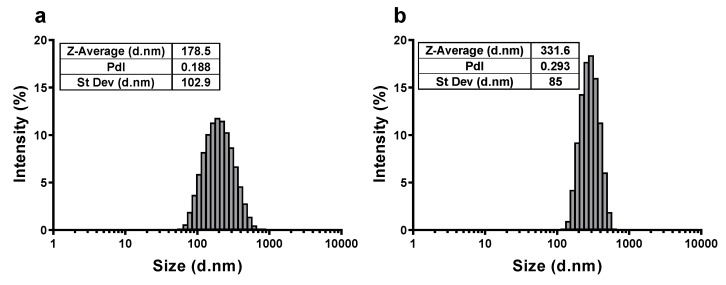
Average size distribution measured by dynamic light scattering: (**a**) GP1 and (**b**) GP2 in 0.02% sodium cholate; PdI: polydispersity index; Z-average: R_h_; St Dev: standard deviation

The morphology characterization, carried out using electron microscopes, is presented in Figure 1 for all C-BNM. Both TEM and SEM images clearly show shape heterogeneity of GP. Both GP form small aggregates, whereas clearly smaller GP1 (80~300 nm of lateral size; Figure 1a–c) form lumps-like flakes indicating a significantly lower quality of this GP. On the other hand, as seen in Figure 1d–f, GP2 form flakes with lateral size about 250~400 nm blade-like edges which could cause damage of intracellular membranes. These results correspond with the average size distribution measured by DLS using Zetasizer (Figure 2). The calculated zeta-average diameters of *R_h_* are 178.5 ± 110 nm and 315 ± 78 nm for GP1 and GP2, respectively. However, as the DLS method is the most suitable for the measurement of spherical particles, therefore the flat shape of GP and presence of aggregates must be considered for the evaluation of these analyses. Figure 1g–i shows the 10 µm long MWCNT with a diameter of 110~200 nm. Detailed physical characterisation (Raman spectra, X-ray diffraction, elemental composition, thermogravimetric analyses) is provided in the Supplementary Material.

### 3.2. Zeta Potential

**Figure 3:**
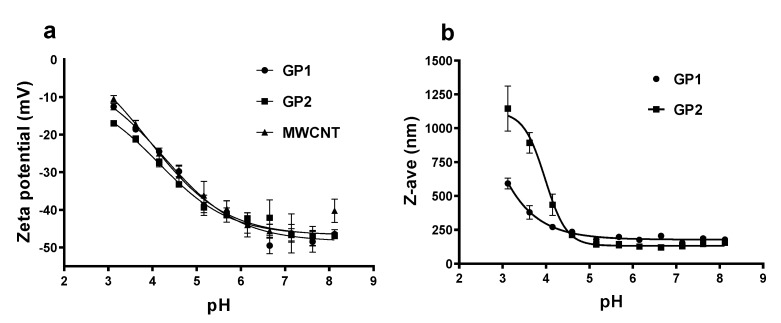
Effect of pH on (**a**) *ζ*-potential of C-BNM in 0.02% sodium cholate; (**b**) average diameter of C-BNM in 0.02% sodium cholate

Figure 3 shows pH-dependence of the (a) ζ potential of C-BNM and (b) aggregation state of GP1 and GP2. Negative ζ potential of C-BNM (*ζ* < -40 mV) in neutral pH corresponds with presence of sodium cholate and residual oxygen (see Supplementary Material) on the surface of these materials. In the pH range 6–8, the aggregation of particles was also significantly less pronounced than in acidic pH. Acidification to pH 3 led to an increase of ζ potential to *ζ* > –20 mV and a considerable increase of aggregation which is reflected by an increase in the average size (Z-average) (Figure 3b). Below pH 4 GP2 exerted formation of significantly larger aggregates in comparison to GP1. Transferring of these well dispersed C-BNM into cell medium with FBS caused formation of biocorona which was reflected by changing an average ζ potential to values: –8.52 mV for GP1; –10.8 mV for GP2; and –13.1 mV for MWCNT.

### 3.3. Intracellular Localization of C-BNM 

**Figure 4:**
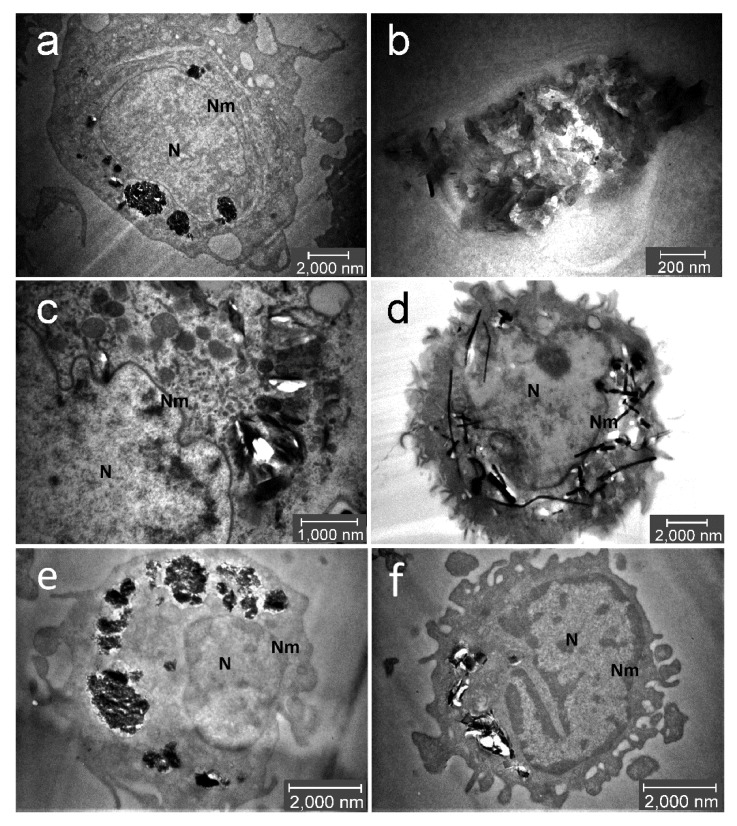
Intracellular localisation of C-BNM after 24 h exposure in THP-1 (**a**–**d**) and primary monocytes (**e**,**f**): GP1 (**a**,**b**,**e**) forms large aggregates in cytoplasm apparently in vesicles. No particles are found in nucleus (N); (**c**,**f**) GP2 forms smaller aggregates in cytoplasm, apparently in vesicles. Occasionally, free particles are detected in cytoplasm; (**d**) MWCNT were found as free needle-like objects in the cytoplasm, possibly from damaged lysosomes. Sporadically, they can be found in nucleus or penetrating through the nuclear membrane (Nm).

TEM confirmed presence of all GP and MWCNT in cytoplasm of THP-1 macrophages after 24 h of exposure (Figure 4). As seen in Figure 4a–c, GP particles were observed in endosomes and no particles were localized in nucleus. GP1 formed large aggregates (Figure 4b) whereas GP2 formed smaller aggregates (Figure 4c) located in vesicles. Moreover, in the case of GP2, free particles were found sporadically in cytoplasm. We observed a similar pattern of GP distribution in human primary monocytes (Figure 4e,f). Further, MWCNT were located as free particles in whole cytoplasm, where they could possibly interact with multiple organelles (Figure 4d). Damaged cell structures suggest an escape of tubes from endosomes or lysosomes as it corresponds with the release of cathepsin B (see Section 3.5. Activation of NLRP3 and Release of Pro-Inflammatory Cytokines). Individual nanotubes were observed to penetrate also through the nucleus membrane.

### 3.4. Cell Viability 

**Figure 5:**
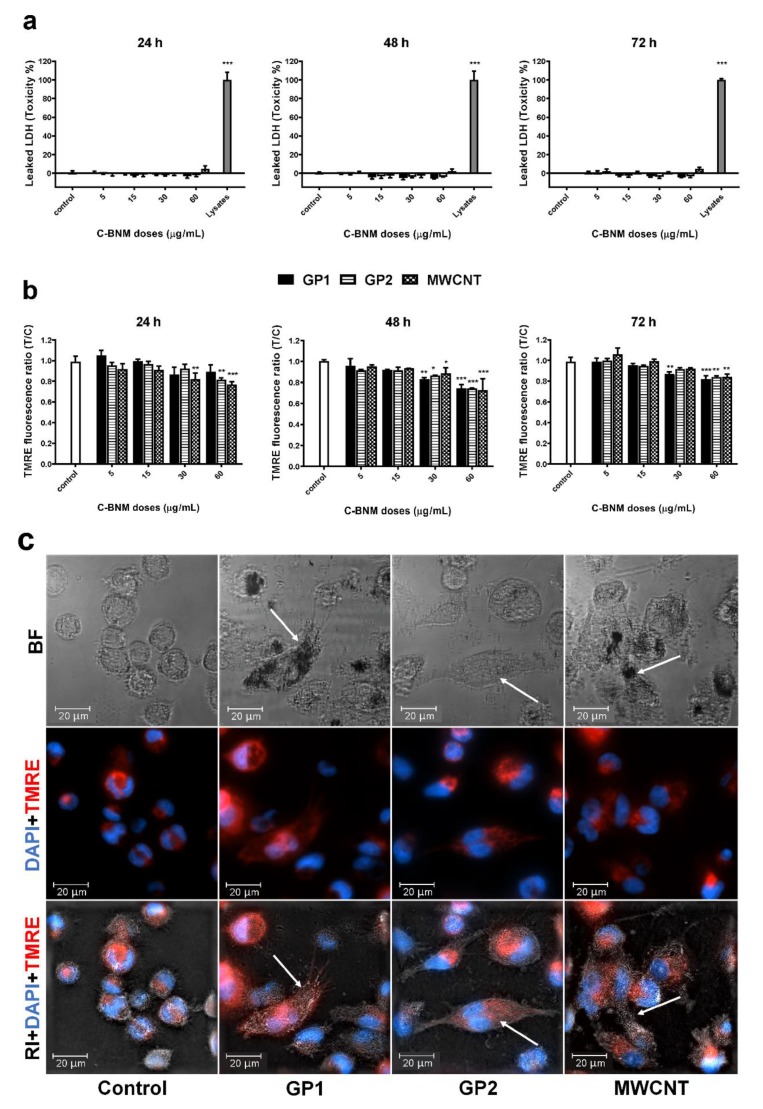
Cell responses to C-BNM: (**a**) Percentage of cytotoxicity via LDH assay after 24 h, 48 h and 72 h exposure. Data are reported as average ± standard error of the mean (*Toxicity % = (T – C)/(L – C) × 100*); *T*—test cells; *C*—untreated control; *L*—lysates. The symbol *** p < 0.001 highlights statistical significance as compared to the corresponding *C*; (**b**) mitochondrial potential via TMRE staining after 24 h, 48 h and 72 h exposure. Data normalised to control (untreated cells) represent an average ± standard error of the mean. The symbols * p < 0.05; ** p < 0.01; *** p < 0.001 highlight the statistical significance as compared to the corresponding control; (**c**) representative images of THP-1 cells loaded with C-BNM (white arrows), after 24 h exposure, with labelled active mitochondria (TMRE) and nuclei (DAPI) detected using a holotomographical microscopy; RI—refractive index; BF—bright field.

Cell viability, assessed with LDH assay, was determined after 24 h, 48 h and 72 h of cell exposure to C-BNM (5–60 µg/mL). Cytosolic enzyme LDH which is released into the cell medium after damage of the plasma membrane during cell death, serves as a well-established and reliable indicator of cellular toxicity. Studied C-BNM did not induce any significant cell membrane damage and subsequent release of LDH into cytoplasm (Figure 5a). We also evaluated the mitochondrial membrane potential via TMRE staining after 24 h, 48 h and 72 h of cell exposure to C-BNM (5–60 µg/mL). In TMRE labelled active mitochondria, a decrease of the fluorescent intensity corresponds with a decrease of the mitochondrial activity or its damage. All C-BNM induced only slight dose dependent decrease (10–20%) in mitochondrial activity (Figure 5b). Moreover, microscopy studies did not reveal any significant mitochondrial damage (Figure 5c). Finally, no statistical difference was observed in the mitochondrial potential decrease, between all types of C-BNM.

### 3.5. Activation of NLRP3 and Release of Pro-Inflammatory Cytokines

**Figure 6:**
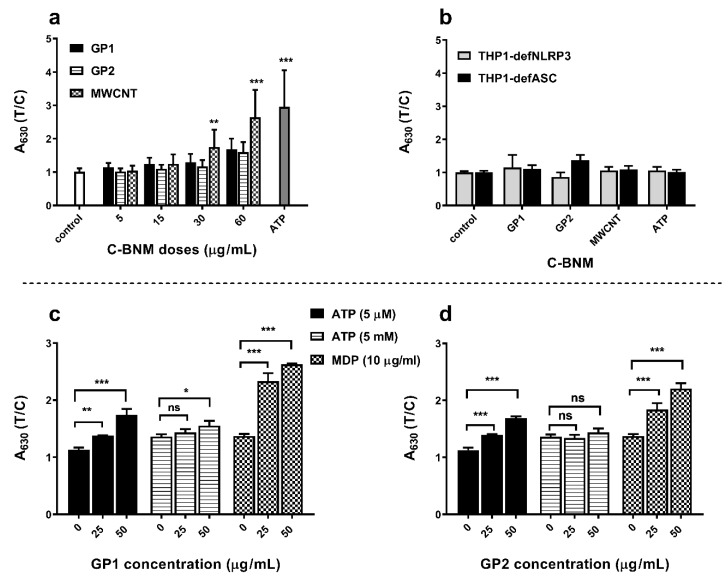
Effect of C-BNM on activation of inflammasome NLRP3 after 24 h exposure: (**a**) NLRP3 activation in THP1 null cells was measured as conversion of proIL-1β to IL-1β, which was detected using HEK-Blue™ IL-1β cells. Data were normalised to the control (untreated THP1-null cells). ATP was used as positive standard of NLRP3 induction. The symbols ** p < 0.01; *** p < 0.001 highlight the statistical significance as compared to the corresponding control; (**b**) Activation of NLRP3 in deficient cells THP1-defNLRP3 and THP1-defASC, which were treated the same way as THP1-null cells; (**c**,**d**) activation of NLRP3 in THP-1 null macrophages by GP in presence of MDP and ATP: ATP (5 mM; 5 µM) and MDP (10 µg/mL) were used as a standard activators of NLRP3 (control) in presence of 0, 25 and 50 µg/mL of GP. The symbols * *p <* 0.05; ** p < 0.01; *** p < 0.001 highlight statistical significance as compared to the corresponding controls (0) without GP.

Activation of inflammasome is a key step in the release of pro-inflammatory cytokine IL-1β. In this study, we measured IL-1β in supernatants of THP1-null cells which represent a model of canonical pathway activation of NLRP3. The cells were exposed to increased concentrations of C-BNM (5–60 µg/mL) and to ATP (5 mM) as a positive control. Results are summarized in Figure 6a. Both GP induced a slight, but statistically not significant increase of IL-1β secretion at the highest dose tested. In contrast to GP, MWCNT were able to activate inflammasome and subsequently release IL-1β in a dose-dependent manner. To observe possible activation of other inflammasomes than NLRP3, we examined supernatants of exposed THP1-defNLRP3 and THP1-defASC, which are deficient in NLRP3 receptor and ASC adaptor protein, respectively. The results confirmed specific activation of NLRP3 by MWCNT (Figure 6b). The effect of PAMP molecules was tested with the muramyl dipeptide. Both GP were able to activate NLRP3 in the presence of muramyl dipeptide (Figure 6c,d).

**Figure 7:**
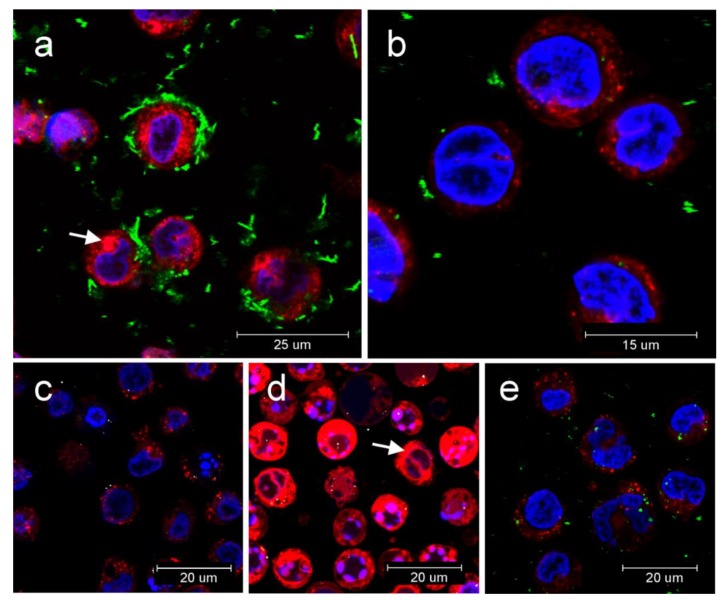
Release of cathepsin B from lysosomes into cytoplasm in THP1-null cell revealed by confocal microscopy: Proteolytic activity of cathepsin B was determined by fluorogenic substrate with red emission; (**a**) release of cathepsin B (red fluorescence) into cytoplasm after 24 h incubation with MWCNT (light scattering in green). Cytoplasm stained with fluorogenic substrate (white arrow); (**b**) Release of cathepsin B after 24 h incubation with GP2 (light scattering in green); (**c**) Negative control; (**d**) release of cathepsin B after incubation with lysosomal disruptor LLME with burst of cathepsin B into cytoplasm (white arrow); (**e**) release of cathepsin B after 24h incubation with GP1 (light scattering in green).

For the evaluation of the possible mechanism of NLRP3 assembly, we measured the release of cathepsin B into cytoplasm using a cell-penetrating fluorogenic substrate (Figure 7). Under normal conditions, this protease is localized in lysosomes. Destabilization of lysosomes by various lysosomal disruptors leads to the release of cathepsin B into the cytoplasm and subsequently the activation of NLRP3 is induced. Figure 7 shows micrographs of C-BNM, visualized by the confocal microscopy in the light scattering mode at wavelengths corresponding to the excitation spectrum of the fluorescent product of CV-(RR)2 substrate cleavage by cathepsin B. The fluorescence was measured after 2 h, 6 h and 24 h after treatment of THP1-null cells with C-BNM. Fluorescent dots represent penetration of a substrate to lysosomes and the disperse signal points to the release of cathepsin B from damaged lysosomes. In addition to the positive control, the most significant release of cathepsin B was observed using MWCNT (Figure 7a), which corresponds to our previous findings on NLRP3 activation (Figure 6a). 

#### Activation of NLRP3 in Isolated Monocytes

**Figure 8:**
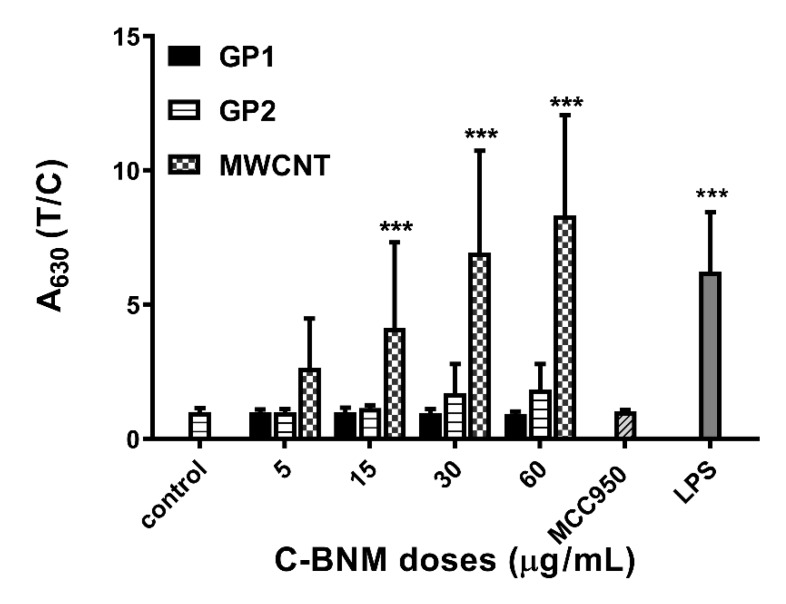
Activation of NLRP3 in isolated monocytes after 24 h exposure was measured as conversion of proIL-1β to IL-1β, which was detected using HEK-Blue™ IL-1β cells. Untreated isolated monocytes were used as a negative control. LPS was used as positive standard of NLRP3 induction. MCC950 was used as specific inhibitor of MWCNT induction of NLRP3. The symbol *** p < 0.001 highlights the statistical significance as compared to the corresponding control.

Verification of NLRP3 activation by an alternative pathway was performed using primary monocytes isolated from human blood. Additionally, in this more complex in vitro model, MWCNT induced strong and concentration-dependent activation of NLRP3 (Figure 8). Application of MCC950 inhibitor confirmed specific activation of NLRP3 inflammasome by MWCNT. When exposed to graphene, only GP2 increased the activity of NLRP3, but without statistical significance when compared to GP1. Viability of monocytes exposed to various C-BNM was confirmed by LDH assay (see supplementary data, Figure S4).

The secretion of the pro-inflammatory cytokines IL-6 and TNFα as well as of the anti-inflammatory cytokine IL-10 was quantified after 24 h and 48 h exposure of THP-1 and primary monocytes to C-BNM and LPS (100 ng/mL) as a positive control. There was no significant release of these cytokines after 24 h or 48 h exposure to all C-BNM (results not shown).

## 4. Discussion

The aim of this study was to evaluate possible proinflammatory and immunomodulatory effects of C-BNM, notably of GP, toxicological data on which are still insufficient. Generally, C-BNM were found to affect complement and all immune cells including macrophages, dendritic cells, lymphocytes, monocytes, neutrophils, eosinophils, NK cells and mast cells. There is no wonder that inflammation is among the general side effects observed by toxicologists after application of C-BNM [41]. Acute or chronic inflammation responses are interfering with the normal physiological functions of important organs [42,43]. C-BNM are known to induce either physical or biological damage to the cell membrane, membranes of organelles along with destabilization of actin filaments, the cytoskeleton and effecting the cell cycle [44,45,46]. At the tissue and cellular levels, the mechanisms responsible for inflammation are based on disruption of various barriers (e.g., alveolar–capillary barrier, blood–brain barrier), infiltration of immune cells and their interaction with molecules released from injured cells (disease associated molecular patterns) or with nanoparticles themselves [47]. A recent study demonstrating destabilisation of phospholipid membranes by carbon nanosheets was published recently by [48].

As inflammasomes play a central role in the process of inflammation we used a well-established model on THP-1 and THP1-null cells to study the key mechanism responsible for the adverse effect of C-BNM. In our previous study we demonstrated the effect of 100 nm nanodiamonds on the injury of lysosomal membranes and the release of cathepsin B resulting in activation of inflammasome NLRP3 and release of IL-1β [6]. Therefore, we used a similar model also for testing of various GP and MWCNT. In this study, we used two types of sterile non-oxidized graphene platelets and commercially available MWCNT to determine their proinflammatory potential. We confirmed the ability of C-BNM to penetrate cytoplasmic membranes and, in the case of MWCNT, nuclear membranes, and accumulate inside the cells (Figure 4). We observed direct stimulation of inflammasome NLRP3 by MWCNT through a release of the proinflammatory cytokine IL-1β (Figure 6). Activation of NLRP3 is a complex process which is evoked in the response to infectious stimuli like whole pathogens or individual microbial components (PAMPs), as well as by cellular stress signals represented by sterile DAMPs. In the canonical pathway, two signals precede the oligomerization of NLRP3. In the case of macrophages/THP1-null model, the first one requires the transcription of inflammasome components including pro-caspase-1 and pro-IL-1β. The second one includes the DAMP/PAMP signals, which are sensed by NLR receptors. In the non-canonical pathway, NLRP3 can be activated through endogenous caspases 4 and 5 (caspase 11 in murine macrophages), which specifically binds lipopolysaccharide from Gram-negative bacteria and triggers misbalance of ions and ATP. ATP then works as an autostimulator of NLRP3 assembly. The result of this stimulus is the cleavage of pro-caspase-1, pro- IL-1β, pro-IL-18 and pro-gasdermin D. Gasdermin D (GSDMD) forms pores in the cell membrane, through which mature IL-1 is released. Increased pore formation together with mitochondrial disbalance may lead to failure of cell homeostasis and, subsequently, to cell death by pyroptosis followed by the leakage of intracellular content, such as LDH [49,50]. 

Neither GP nor MWCNT induced a significant release of LDH even after 72 h incubation (Figure 5a). It means that short term cytotoxicity via pyroptosis was not a direct effect of GP and MWCNT. A slight decrease of mitochondrial potential was observed for all tested materials at concentrations above 30 µg/mL (Figure 5b). These data are in a good accordance with published observations regarding C-BNM as summarised by [51].

ELISA assays confirmed the absence of proinflammatory cytokines IL-6 or TNFα for all C-BNM used in the study. Release of these cytokines is typical of pyrogenic stimulation, so these data confirm the absence of LPS or another PAMPs on the surface of nanomaterials. On the other hand, we observed a proinflammatory potential of MWCNT via activation of NLRP3. While the NLRP3 serves as a sensor of DAMPs and (in the case of macrophages), its own assembly does not depend only on stimulation with LPS (unlike the alternative pathway), making it the most suitable tool for the evaluation of proinflammatory potential [40]. According to images from TEM (Figure 4) we assumed that especially carbon nanotubes may cause a nonspecific intracellular damage of membrane structures, which we confirmed by the detection of Cathepsin B release from the damaged lysosomes (Figure 7a). Cathepsin B is considered as one of the DAMPs sensed by NLR and its presence in cytoplasm leads to NLRP3 oligomerization [6,52]. Interestingly, according to TMRE staining, we detected only a slight decrease of the mitochondrial potential. It can be assumed that mitochondrial damage did not play a pivotal role in inflammasome activation in THP1-null macrophages. However, it correlates with our observation of cell viability even after 72h after stimulation with C-BNM and rejects the possibility of non-canonical activation of inflammasome, which usually leads to pyroptosis through misbalance of ATP [49,53]. This ability to secrete IL-1β while retaining viability is similar to the situation in primary monocytes, whose NLRP3 (Figure 8) is activated via an alternative pathway, and also has been described in human bone marrow derived macrophages as a hyperactivation state [50,54]. It has been proven that several self-derived DAMPs as isolated lipid components, like self-encoded oxidized phospholipids (oxPAPC), led to the GSDMD-dependent release of IL-1β without cell death and LDH release [54]. Participation of other inflammasomes (e.g., AIM2, NLRC4, NLRP1, etc.) was excluded by using specific THP-1 macrophages deficient in NLRP3 and ASC in our study (Figure 6b). 

It has been reported that oxidized forms of graphene and nanotubes caused the release of proinflammatory cytokines IL-6, TNFα and IL-8 through oxidative stress as a main mechanism [15,17]. On the other hand, pristine graphene without specific carboxyl or hydroxyl groups should not specifically interact with pattern recognition receptors (PRR) on the surface of cells. Thus, its potential cytotoxicity strongly depends on its shape and size [30,55]. Unlike MWCNT, GP were found enclosed as smaller (GP2) or bigger (GP1) aggregates in endosomes (Figure 4). We detected either no or only slight release (GP2) of cathepsin B (Figure 7) to cytoplasm and together with the results from LDH assay and TMRE staining (Figure 5) it corresponded with a slight, nonsignificant release of IL-1β (Figure 6a). Autophagy, which inhibits NLRP3 activation, could be a possible reason and has been referred to as a common phenomenon in graphene-focussed studies [56]. Generally, despite the different character of used GP, neither of them alone was able to activate NLRP3 to a significant level. A quite different situation occurred when muramyl MDP was added as the prominent representative of DAMP. There was a significantly stronger activation of NLRP3 by both GP1 and GP2 together with MDP than in the case of using MDP alone (Figure 6c,d). These results relate to the contention that GP and MWCNT may serve as a “Trojan horse” and inhaled particles of GP or MWCNT may carry contaminants on its surface. It must be considered that NLRP3 strongly responses to accumulated signals, and it is possible that “harmless” nanomaterial may boost a stimulus from adsorbed PAMPs/DAMPs. There is also an important fact that C-BNM are considered not fully biodegradable materials which are facing the continuous surveillance of the immune system. Therefore, although not acutely toxic, it may cause chronic problems under long-term exposure, owing to accumulation in tissues [43,57]. If the accumulation reaches the certain threshold, the second signals like DAMP or PAMP originating from damages can trigger activation of inflammasome. On the other hand, recent studies suggested a possibility of partial degradation of GP and MWCNT by macrophages vie enzymes like myeloperoxidase. This means that inflammatory mechanisms are inevitably involved in the elimination of C-BNM [58].

THP1-null cells represent a pure model to study activation of NLRP3 inflammasome and the proinflammatory potential was confirmed in the case of both GP and especially MWCNT. The data obtained on THP1-null cells were verified on peripheral blood monocytes which represent a model closer to the realistic in vivo scenario and possibility of activation of NLRP3 via an alternative pathway. Contrary to THP-1 null cells, one must consider possible genetic variations among the healthy volunteers from whom the monocytes were isolated. Nevertheless, both models convincingly pointed to a proinflammatory potential of C-BNM, especially MWCNT. An example is the study in which the pulmonary exposure of MWCNT in mice not only led to local inflammation, but also promoted systemic inflammation and systemic inflammation together with dysfunction of the NOS system [59].

## 5. Conclusions

In our study using a well-established in vitro model, we have demonstrated a clear proinflammatory potential of GP and MWCNT which can be enhanced by various PAMP. Therefore, it must be considered that, in in vivo conditions, accumulation of PAMPs and DAMP signals can act synergistically with nanomaterials, even if they are considered “harmless” on their own. Such a synergic action can lead to activation of inflammatory mechanisms, e.g., via NLRP3. There is also an important fact that graphene as well as CNT are not fully biodegradable materials and, therefore, although rendered not acutely toxic due to various surface modifications, they may cause chronic problems under long-term exposure owing to accumulation in tissues and organs. Questions regarding the effects of C-BNM accumulated for instance, in brain, lungs, liver or spleen in the course of real infection (e.g., influenza, hepatitis or EBV) of these organs are of additional importance. Studies focused on aspects of interaction between C-BNM and organisms at the molecular, cellular, tissue and whole body level are necessary to fully understand mechanisms of toxicity and to evaluate possible risks imposed by such materials to humans and the environment, if broad application of C-BNM were accomplished. Therefore, GP and MWCNT may serve as a “Trojan horse” and inhaled particles of C-BNM in real environment may carry contaminants on its surface, which can have a nature characteristic of PAMPs.

## Supplementary Materials

The following are available online at https://www.mdpi.com/2079-4991/10/3/418/s1. Figure S1: XRD patterns of studied C-BNM. Table S1: Elemental composition of C-BNM. Figure S1: Raman spectra of MWCT, GP1 and GP2. Figure S3: TGA (left Y-axis) and TGA (right Y-axis) curves recorded for C-BNM under oxygen atmosphere with a heating rate of 10 K/min. Figure S4: Monocytes response to C-BNM; Percentage of cytotoxicity via LDH assay after 24 and 48 h.
