# Supplementary material for: Proinflammatory Effect of Carbon-Based Nanomaterials: In Vitro Study on Stimulation of Inflammasome NLRP3 via Destabilisation of Lysosomes
Source: Nanomaterials (Basel). 2020 Feb 27;10(3):418. doi: 10.3390/nano10030418 (PMC7152843; doi:10.3390/nano10030418)
Supplement: Supplementary file 1 [file nanomaterials-10-00418-s001.pdf]

Supplementary Information

Proinflammatory Effect of Carbon-Based Nanomaterials: In Vitro Study on Stimulation of Inflammasome NLRP3 via Destabilisation of Lysosomes

Tereza Svadlakova [1,2], Frantisek Hubatka [3], Pavlina Turanek Knotigova [3], Pavel Kulich [3], Josef Masek [3], Jan Kotoucek [3], Jan Macak [4], Martin Motola [4], Martin Kalbac [5], Martina Kolackova [1], Radka Vankova [1], Petra Vicherkova [1], Andrea Malkova [2], Pavlina Simeckova [3], Yuri Volkov [6,7], Adriele Prina-Mello [6], Irena Kratochvilova [8], Zdenek Fiala [2], Milan Raska [3,9], Jan Krejsek [1,*] and Jaroslav Turanek [3,*]

1 Institute of Clinical Immunology and Allergology, University Hospital Hradec Kralove and Faculty of Medicine in Hradec Kralove, Charles University, 50005 Hradec Kralove, Czech Republic; svadlakovat@lfhk.cuni.cz (T.S.); kolackovam@lfhk.cuni.cz (M.K.); vankovr@lfhk.cuni.cz (R.V.); petraavicherkova@gmail.com (P.V.)
2 Institute of Hygiene and Preventive Medicine, Faculty of Medicine in Hradec Kralove, Charles University, 50003 Hradec Kralove, Czech Republic; Malka8AR@lfhk.cuni.cz (A.M.); fiala@lfhk.cuni.cz (Z.F.)
3 Veterinary Research Institute, 62100 Brno, Czech Republic; hubatka@vri.cz (F.H.); knotigova@vri.cz (P.T.K.); kulich@vri.cz (P.K.); masek@vri.cz (J.M.); kotoucek@vri.cz (J.K.); simeckova@vri.cz (P.S.); milan.raska@upol.cz (M.R.)
4 Center of Materials and Nanotechnologies, Faculty of Chemical Technology, University of Pardubice, 53002 Pardubice, Czech Republic; Jan.Macak@upce.cz (J.Ma.); martin.motola@upce.cz (M.M.)
5 J. Heyrovsky Institute of Physical Chemistry of the Czech Academy of Sciences, 18223 Prague, Czech Republic; martin.kalbac@jh-inst.cas.cz (M.Ka.)
6 Department of Clinical Medicine/Trinity Translational Medicine Institute (TTMI), Trinity College Dublin, D08 W9RT, Dublin, Ireland; yvolkov@tcd.ie (Y.V.); prinamea@tcd.ie (A.P.M.)
7 Department of Histology, Cytology and Embryology, First Moscow State Sechenov Medical University, 119992, Moscow, Russian Federation
8 Institute of Physics, Czech Academy of Sciences, 18200 Prague, Czech Republic; krat@fzu.cz (I.K.)
9 Department of Immunology and Institute of Molecular and Translational Medicine, Faculty of Medicine and Dentistry, Palacky University Olomouc, 77515 Olomouc, Czech Republic
* Correspondence: jan.krejsek@fnhk.cz (J.K.); turanek@vri.cz (J.T.)

Received: 07 February 2020; Accepted: 24 February 2020; Published: date

XRD

Experimental

The X-ray diffraction patterns (XRD) were measured on Panalytical Empyrean diffractometer using a Cu X-ray tube and scintillation detector Pixcel^{3D} (ThermoFisher Scientific, Waltham, MA, USA). The measurements were performed in the 2θ range of 20 – 40°, the step size was 0.026°. The diffractometer was equipped with a *xyz* programmable stage.

Results

Figure S1 shows the XRD patterns of studied C-BNM. In the case of MWCNT, the peak at 2θ ~26.3°with a d-spacing value of 3.38 Å is assigned to the (002) plane of the hexagonal graphite structure. In both GP1 and GP2, the peak centered at 2θ ~26.4°with d-spacing value of 3.36 Å is assigned to the (002) plane of the hexagonal structure. A dramatically decrease in the intensity of GP1 might be caused by the decrease of thickness of graphite due to the break of inter-planar carbon within graphite structure during the preparation method.

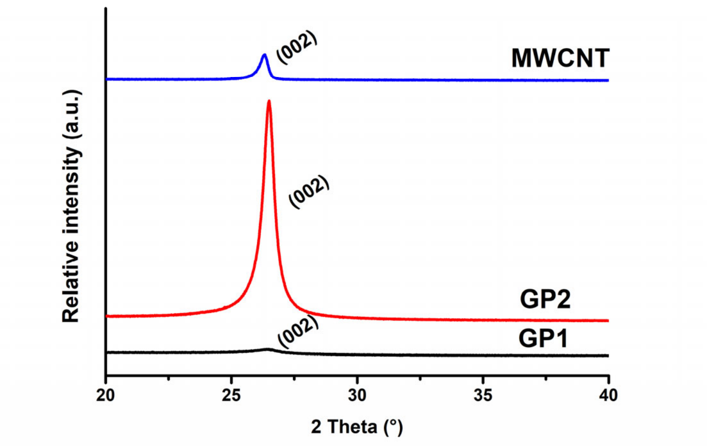

Figure S1. XRD patterns of C-BNM

Elemental composition

Elemental compositions of C-BNM were analysed using Energy dispersive X-ray (EDS) analyses by mapping of surface area of 200 x 200 mm at 20 kV using EDS detector (AZtec X-Max 20, Oxford Instruments, Abingdon, UK) integrated with SEM.

	**Elemental composition**	
**Sample**	**C (at %)**	**O (at %)**
GP1	92.5	7.5
GP2	95.5	4.5
MWCNT	98.5	1.5

Table S1. lists the elemental composition of all C-BNM obtained by EDS. As it can be seen, for all materials, EDS revealed the presence of carbon and oxygen. The obtained results are in a good agreement with the product description of manufacturers-providers.

Raman spectroscopy

Experimental

Raman measurements were performed using a LabRAM HR Raman spectrometer (Horiba Jobin–Yvon, Kyoto, Japan) with laser excitation wavelength of 633 nm and a laser power of 1mW under the 100x objective to avoid heating of the sample.

Results

Figure S2 shows Raman spectra of studied C-BNM, characterised by the occurrence of the following bands:

- ~1350 cm^{-1}; D band is indicative of impurities and/or structural disorder in the C-BNM sample.
- ~1580 cm^{-1}; G band is the result of C-C bond stretching. The G band is sensitive to doping and both the line width and frequency of this peak can be employed to check the doping level.

- ~2685 cm^{-1}; 2D band corresponding to stresses. The lineshape and width of the 2D mode can be in some cases used to estimate the number of AB stacked graphene layers.

For all studied C-BNM the strong D band indicate the presence of t significant amount of defects. The D mode intensity differs and it determine the disorder in the sample i.e. higher the intensity of D band means increased number of disorder/defects in sample

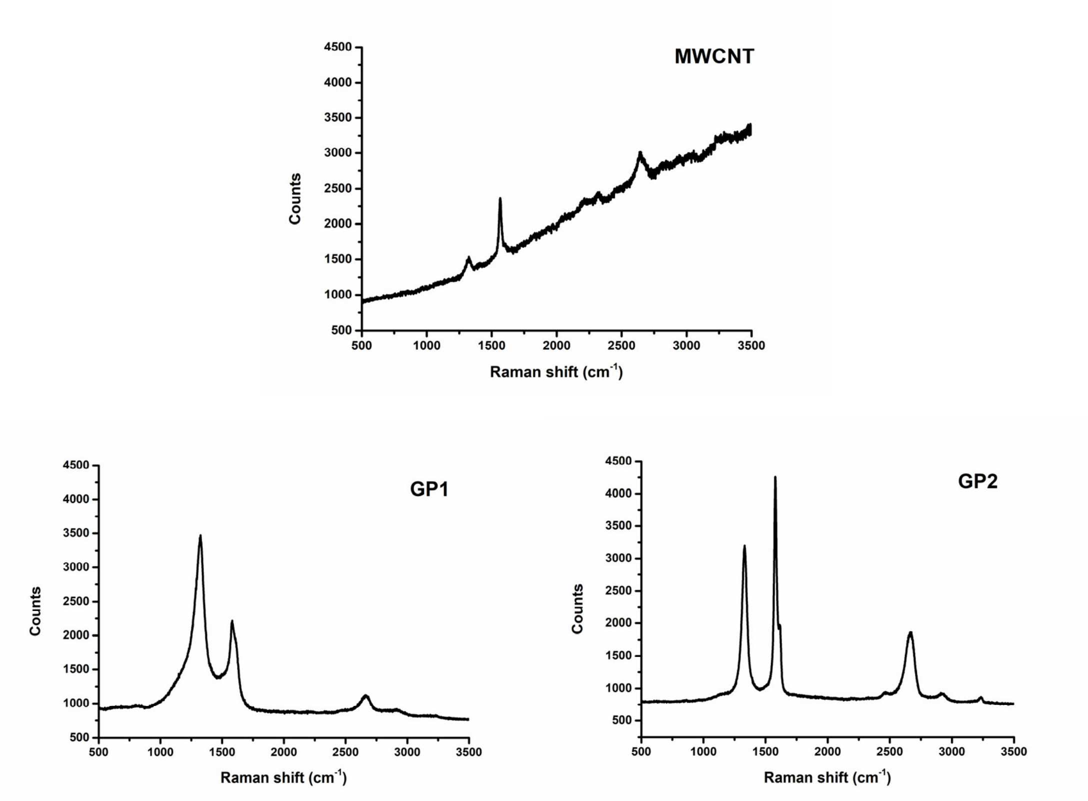

Figure S2. Raman spectra of MWCT, GP1 and GP2

Thermogravimetric analyses

Experimental

Thermal decomposition of C-BNM was studied using Differential Scanning Calorimetry (DSC) and Thermogravimetric Analysis (TGA) on STA 449 F1 Jupiter® NETZSCH (Selb, Germany) to determine the combustion temperature and weight loss. Measurements were performed under oxygen atmosphere with a heating rate of 10 K/min.

Results

Figure S3A-C shows TGA and DSC results

A) MWCNT
The oxidation of MWCNT occurred between 700 – 800 °C
DSC scan has an endothermic peak at ~770 °C which is associated with the thermal combustion of the nanotubes. From the TG scan, at ~800 °C, the sample burned up with a zero residual mass.

B) GP1
The oxidation of GP_1 occurred between 400 – 700 °C
DSC scan has an endothermic peak at ~650 °C which is associated with the thermal decomposition/combustion of graphene. From the TG scan, at ~700 °C, the sample burned up

with a residual mass of 6%. This means that ~94% weight loss accompanies the endothermic DSC peak.

C) GP2

The oxidation of GP2 occurred between 300 – 600 °C

DSC scan has an endothermic peak at ~565 °C which is associated with the thermal decomposition/combustion of graphene. From the TG scan, at ~670 °C, the sample burned up with a residual mass of 2%. This means that ~98% weight loss accompanies the endothermic DSC peak. The two peaks at ~310 °C and ~400 °C are presumably associated with the combustion of amorphous carbon.

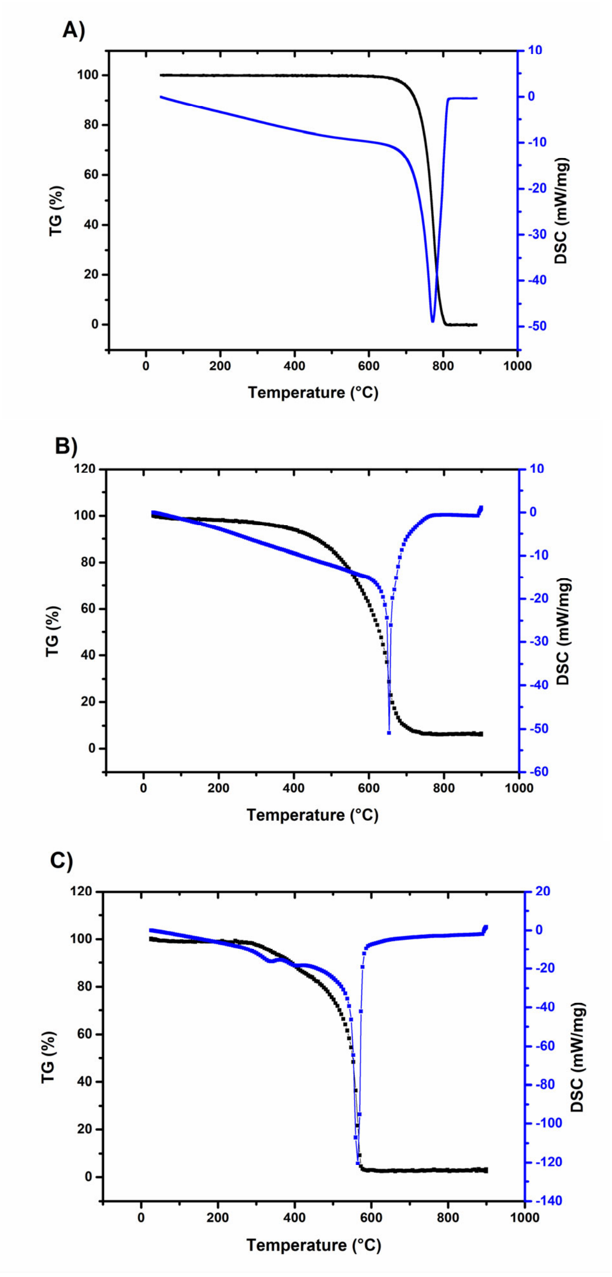

Figure S3. TGA (left Y-axis) and TGA (right Y-axis) curves recorded for C-BNM under oxygen atmosphere with a heating rate of 10 K/min

Cell viability and plasma membrane integrity of isolated monocytes

Experimental

Cell viability was assessed through lactate dehydrogenase (LDH) assay. Isolated monocytes were seeded in flat bottom 48-well plate at density 1.5 x 10^5 cells per well and exposed to increasing concentration of GP and MWCNT in media (5 – 60 μg/mL) for 24 – 48 h. Cells with no exposure and cells exposed to sodium cholate were used as controls. Supernatants were centrifuged for 10,000x g for 10 min to get rid of GP and MWCNT and transferred in new flat bottom 96-well plate. The LDH assay was performed according to the manufacturer's protocol. Absorbance was measured in a microplate spectrophotometer Synergy HTX (Biotek, Bad Friedrichshall, Germany) at 490 nm, with 690 nm set as the reference wavelength.

Results

Cell viability was determined after 24h and 48h of cell exposition to C-BNM (5 – 60 μg/ml). Studied GP did not induce any significant cell membrane damage and subsequent release of LDH into cytoplasm. MWCNT caused release of LDH (~ 10 – 15%) from damage cell membrane only at higher dose tested (60 μg/ml) after 24 h and 48 h (Figure S4).

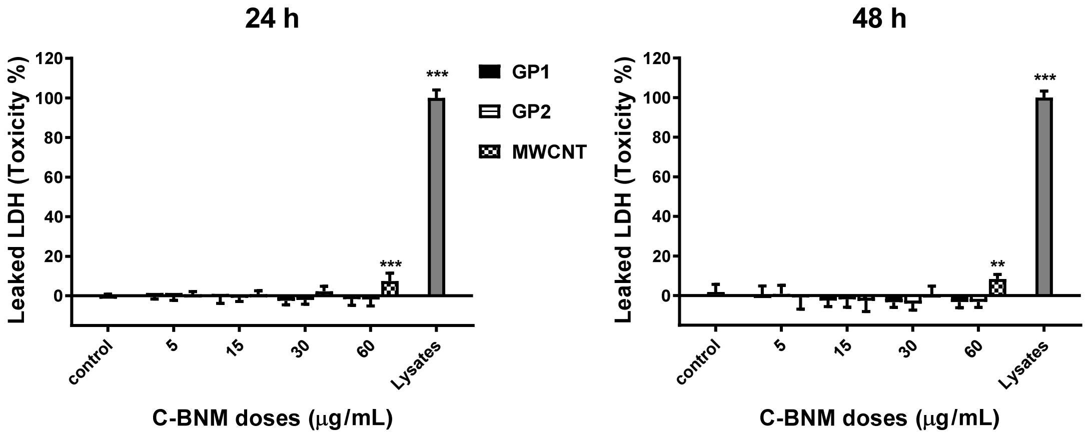

Figure S4. Monocytes response to C-BNM; Percentage of cytotoxicity via LDH assay after 24 and 48h. Data are reported as average ± standard error of the mean (*Toxicity % = (T-C)/(L-C)*100*), *T* – test. cells, *C* – untreated control, *L* – Lysates; the symbol *** *P* <0.001 highlights statistical significance as compared to the corresponding C.
